# Ethnic differences and parental beliefs are important for overweight prevention and management in children: a cross-sectional study in the Netherlands

**DOI:** 10.1186/1471-2458-12-867

**Published:** 2012-10-12

**Authors:** Paul L Kocken, Yvonne Schönbeck, Lidewij Henneman, A Cecile JW Janssens, Symone B Detmar

**Affiliations:** 1 Department of Child Health, TNO, P.O. Box 2215, Leiden, 2301 CE, The Netherlands; 2 Department of Public and Occupational Health, Department of Clinical Genetics, VU University Medical Center Amsterdam, EMGO Institute for Healthcare Research, P.O. Box 7057, Amsterdam, 1007 MB, The Netherlands; 3 Department of Public Health, Erasmus University Medical Center Rotterdam, P.O. Box 2040, Rotterdam, 3000 CA, The Netherlands; 4 Department of Public Health and Primary Care, Leiden University Medical Center (LUMC), P.O. Box 9600, Leiden, 2300 RC, The Netherlands

**Keywords:** Child obesity, Overweight, Culture, Health promotion

## Abstract

**Background:**

The prevalence of obesity and overweight is highest among ethnic minority groups in Western countries. The objective of this study is to examine the contribution of ethnicity and beliefs of parents about overweight preventive behaviours to their child’s outdoor play and snack intake, and to the parents’ intention to monitor these behaviours.

**Methods:**

A cross-sectional survey was conducted among parents of native Dutch children and children from a large minority population (Turks) at primary schools, sampled from Youth Health Care registers.

**Results:**

Native Dutch parents observed more outdoor play and lower snack intake in their child and had stronger intentions to monitor these behaviours than parents of Turkish descent. In the multivariate analyses, the parents’ attitude and social norm were the main contributing factors to the parental intention to monitor the child’s outdoor play and snack intake. Parental perceived behavioural control contributed to the child’s outdoor play and, in parents who perceived their child to be overweight, to snacking behaviour. The associations between parents’ behavioural cognitions and overweight related preventive behaviours were not modified by ethnicity, except for perceived social norm. The relationship between social norm and intention to monitor outdoor play was stronger in Dutch parents than in Turkish parents.

**Conclusions:**

As the overweight related preventive behaviours of both children and parents did differ between the native and ethnic minority populations of this study, it is advised that interventions pay attention to cultural aspects of the targeted population. Further research is recommended into parental behavioural cognitions regarding overweight prevention and management for different ethnicities.

## Background

Overweight and obesity have increased worldwide. In 2010, more than one in ten of the world’s adult population was obese [1]. Being overweight as a child increases the risk of being overweight as an adult [2,3]. Childhood overweight and obesity have detrimental effects on both physical and mental health of children, including increased risk for high cholesterol, high blood pressure, type 2 diabetes [4], social discrimination and low-self-esteem [5]. There is therefore general agreement that it is important to start overweight prevention at an early age. Repeating international evidence exists that in western countries prevalences of obesity and overweight are highest among ethnic minority groups [6-9]. In Dutch children from Turkish origin, prevalence rates are two to three times higher than in autochthonous Dutch children [10,11]. A recent study found that the prevalence of overweight and obesity increases faster among Turkish children than among native Dutch children [12].

Obesity and overweight in minority and social disadvantaged groups may be explained by their lack of resources, both economical and knowledge, which reduces their ability to control weight gain through making healthy food choices and taking opportunities for physical activity [6,13,14]. Moreover, dietary and physical activity patterns influenced by cultural and religious norms, and cultural opinions about body shape and acceptable weight gain, may explain the increase in overweight and obesity in certain populations [15-17]. Considering the large difference in overweight prevalence between ethnic groups, it is expected that overweight preventive behaviours and related perceptions differ between these groups. The influence of the child’s family is a key factor in overweight prevention [18]. The ethnicity of parents and their beliefs about overweight preventive behaviours in children will be important for interventions, targeting overweight prevention and weight management in children.

Interventions are aimed at dietary behaviour, physical activity (PA) and sedentary behaviour. So far, many studies focus on the relationship of parenting practices with child overweight and parental misperceptions of the child’s weight [2,16,19-21]. To our knowledge, comprehensive studies of ethnic differences in parental behavioural cognitions regarding the food intake and physical activity of their preadolescent children are less common. Behavioural cognitions, i.e. intention, attitude, perceived social norm and behavioural control, from the Theory of Planned Behaviour (TPB), in general predict health behaviours in individuals [22]. Some studies found that overweight risk behaviours in children are associated to less behavioural control in parents [23] and healthy food intake was associated with parental encouragement [24]. However, insight in differences in parental cognitions between ethnic groups is lacking. Cullen et al. found only few differences between parents and children of ethnic groups with regard to cognitions toward dietary behaviours [25]. Also little is known about the importance of parental beliefs about genetic susceptibility for overweight [26]. Parents who think overweight is inherited may be less determined to take action against overweight, than parents who believe overweight is related to over-eating and little physical activity [27].

In this article, parental beliefs about factors influencing overweight preventive behaviours of their child are studied in a native Dutch population and a population of Turkish descent. Turkish people are the largest minority group in the Netherlands. Differences in the contexts underlying the behaviours of ethnic groups are relevant for the development of culturally sensitive preventive interventions [15,28,29]. Resnicow et al. make a distinction between interventions and materials that fit in the language and cultural aspects of the target group (“surface structure”), and psychological and social-cultural factors that determine the behaviour of ethnic groups (“deep structure”) [28]. This raises the question whether both superficial aspects of intervention delivery and deeper determinants of behaviour are relevant for changing overweight preventive behaviours of the native Dutch and ethnic Turkish group.

The following research questions were addressed: How do parental beliefs about overweight preventive behaviours contribute to their child’s outdoor play and snack intake, and to the parents’ intention to monitor these behaviours? How is ethnicity related to these child’s overweight related preventive behaviours and parental intentions?

## Methods

### Subjects and procedures

A cross-sectional survey was conducted to collect information from parents about overweight prevention and management for their children. Autochthonous Dutch children and children of Turkish descent, were selected from the Youth Health Care registers of two Regional Health Services in the Netherlands. Children who had a routine medical examination from Youth Health Care physicians and nurses between September 2005 and December 2006, and who were born between 1 January 2000 and 30 September 2000, or between 1 January 1995 and 30 September 1995, were eligible for the study group. The registers were sorted by date of birth. All the Turkish children who met the criteria were selected. The first two Dutch autochthonous children after each Turkish child in the register were also selected. A child was defined as Turkish when at least one parent was born in Turkey. A child was defined as autochthonous Dutch, when both parents were born in the Netherlands. Postal questionnaires were sent out to the parents of the selected children. The parents of Turkish children received a questionnaire in Dutch and a translated questionnaire in Turkish. They were free to choose which questionnaire to return. The parent who spent most time with the child was asked to fill out the questionnaire. Two reminders were sent to non-respondents. On average, the time period between the medical examination and survey was eight months. The respondents were asked to give informed consent to combine data about the child’s height and weight from the Youth Health Care registers with data collected with the questionnaire. The study protocol was approved by the internal TNO Review Board.

### Behavioural model

Two overweight preventive behaviours were the central outcome measures in this study: the child’s activity and feeding behaviour, i.e. outdoor play and snacking behaviour. The parental intentions to monitor these behaviours were outcome measures also. The difficulties parents had in monitoring the outdoor play and snacking behaviour of their child emerged from qualitative focus group interviews with three groups of Dutch and three groups of Turkish parents that were held during the preparation stage of the survey (unpublished data). Therefore these behaviours were expected to apply to most Turkish and Dutch parents and were included as outcome measures.

The behaviours that contribute to the development of overweight and obesity are influenced by social-cognitive determinants described in the TPB. According to the TPB, intention is the strongest predictor for performing a specific behaviour. Intention is determined by attitude, subjective norm and perceived behavioural control. In this study, the association of the central behaviours child’s outdoor play and snacking behaviour and parental intentions to monitor these behaviours with the TPB determinants were examined [22]. External factors relevant for behaviours related to overweight and obesity in children, i.e. ethnicity, socio-economic status of the parent, the child’s age, the child’s overweight status, and parental opinions about the genetic causes of overweight were considered to be distal determinants [30]. These distal factors may affect overweight preventive behaviour through the proximal TPB-determinants of behaviour.

### Questionnaire

The questionnaire included standard measures derived from questionnaires used in previous studies, such as scales based on determinants of behaviour change taken from the Theory of Planned Behaviour (Table 1) [31]. The questionnaire was pre-tested with parents of both Dutch and Turkish ethnicity.

**Table 1:** Characteristics of scales and items in the questionnaire

**Scale/item**	**# items**	**Cronbach’s α/Pearson’s coefficient (r)**	**Examples of items, answer categories and score range**
***Outcomes***			
Child’s snacking behaviour	4	-	How many days a week does your child eat snacks (crisps, cheese cubes etcetera or sweets)? Never (0) – every day (7). How many portions (crisps, cheese cubes etcetera or sweets) does your child eat per day? 1-2 portions a day (1) – more than 4 portions a day (3)
Child’s outdoors playing	2	-	How many days a week does your child play outdoors (outside school time) Never (0) – every day (7). How long does your child generally play outdoors? Less than half an hour per day (1) – more than 3 hours per day (5).
Intention snack intake	1	-	For the next six months, I plan to make sure my child does not eat too many sweets and snacks. Certainly not (-2) – certainly (2)^a^
Intention playing outdoors	1	-	Do you plan to be alert to see that your child plays outdoors enough to prevent or diminish overweight in the next six months? Certainly not (-2) – certainly (2)
***Distal factors***			
Beliefs about genetic causes of overweight	8	α =0.75	A person’s physique is hereditary. Totally disagree (-2) – totally agree (2)
Perceived child weight	2	r = 0.83	What do you think of your child’s weight at this moment? Compared to other children of his/her age, I find my child…. Far too heavy (-2) – far too light (2)
Monitoring (CFQ)^b^	3	α = 0.91	How much do you keep track of the sweets (candy, ice cream, cream cake) that your child eats? Never (-2) – Always (2)
***TPB factors***			
Attitude snack intake	6	α = 0.61	I think it’s alright to watch my child at all time so he/she does not eat too many sweets and snacks. Totally disagree (-2) – totally agree (2)
Subjective social norm snack intake	3	α = 0.85	My partner thinks that I should be alert and see that my child does not eat too many candies and snacks. Certainly not (-2) – certainly (2)
Perceived behavioural control snack intake	2	r = 0.42	Do you think that you will succeed in continuing to watch your child so he/she does not eat too many sweets and snacks if he/she keeps asking for snacks. Certainly not (-2) - certainly (2)
Attitude playing outdoors	6	α = 0.45	If I make sure my child plays outdoors enough, he/she will not become/be less overweight. Totally disagree (-2) – totally agree (2)
Subjective social norm playing outdoors	3	α = 0.91	My parents think that I should be alert and make sure my child plays outdoors enough. Certainly not (-2) – certainly (2)
Perceived behavioural control playing outdoors	2	r = 0.50	Do you think that you will succeed in getting your child to play outdoors enough if he/she wants to watch TV and he/she keeps on at you? Certainly not (-2) - certainly (2)

^a^ The response categories of TPB-items, and the items of the scales perceived child weight and beliefs about genetic causes of overweight, comprised five options with a neutral option in the middle, except for attitude items which had ‘slightly agree’ as middle response option.

^b^Subscale of Child Feeding Questionnaire (CFQ) [33].

The outcome measure child snacking behaviour was assessed using standard questions about days per week and portions per day for snacks. Child physical activity was measured using questions about days per week and hours per day spent at playing outdoors [32]. The outcome measures parental intention to monitor the outdoor play of the child and intention to monitor the snacking behaviour, were developed on the basis of the qualitative focus group interviews with groups of Dutch and Turkish parents prior to the survey.

As to the independent variables of the questionnaire, the questions on beliefs about the TPB determinants of parental behaviours to prevent and manage overweight, i.e. attitudes, subjective social norms, and perceived behavioural control, were also developed partly on the basis of the qualitative focus group interviews [31]. Parenting style with regard to child feeding was measured using the scale monitoring unhealthy eating behaviour from the Child Feeding Questionnaire [33]. A scale for beliefs about genetic causes of overweight was developed for this research, using statements taken from the qualitative interviews. The child’s Body Mass Index (BMI) was derived from the Youth Health Care health record of routine physical examinations. BMI cut-off points for overweight and obesity were defined according to the international obesity task force thresholds [34]. SD scores for BMI were based on the Dutch general population [35]. In addition to BMI from the Youth Health Care health records, parents’ subjective assessment of their child’s weight was obtained. Furthermore, the questionnaire included the following background characteristics: sex, age and country of birth of the child and parents, and socio-economic status, i.e. the educational level of the parent who filled out the questionnaire.

### Analyses

The differences between the response groups of native Dutch and Turkish descent in background characteristics and in their beliefs about overweight prevention or management were explored, using Chi-square tests for categorized variables, t-tests for normally distributed variables and Mann–Whitney tests for skewed variables. Scale scores were assessed using factor and reliability analyses. Total scale scores were calculated, with higher scores being more favorable. The association between the outcome measures parents’ intentions and children’s behaviours, and distal and TPB factors was analysed, using univariate Pearson’s correlation coefficients. Hierarchical multiple linear regression analysis was used to analyse the contribution of distal and TPB factors to the outcome measures. Four separate models were constructed: one for parental intention to monitor outdoor play, one for parental intention to monitor snack intake, one for the reported child’s outdoor play and one for the reported child’s snack intake. Variables that were significantly correlated to the parental intention and child’s behaviour in the univariate analyses were included in the model. In the first step of the regression analysis, distal factors such as parental characteristics and beliefs about genetic causes of overweight were entered into the model using the enter method. In the second step, TPB factors were added. In the last step, the interactions of ethnicity and perceived child weight with the TPB factors were studied. The interaction effects of ethnicity and perceived child weight were included in the analyses, because these risk indicators were of primary interest in this study. We interpreted the interaction effects by inspecting plots [36]. SPSS Statistics 17.0 for Windows was used for the analyses.

## Results

### Response and characteristics of response groups

A total of 882 out of 1617 questionnaires were returned, a response rate of 55%. The response from parents of Dutch children was 57%, and 48% from parents of Turkish children. More than ninety percent of the questionnaires for Turkish children were completed by the parent of Turkish descent. The other parents had different origins. On average, the questionnaire was filled out eight months after the routine health examination. The respondents did not differ from non-respondents in terms of child age and sex. Half the children whose parents answered questions were boys and half were girls (Table 2). The ethnicity of the children was 71% Dutch and 29% Turkish. According to international BMI cut-offs, 11.8% of the children were overweight and 3.9% were obese. Overweight and obesity were considerably higher among the Turkish children than among the Dutch (31.5% and 10.1% respectively). The overweight and obesity prevalences concur with other population studies in the Netherlands [10-12]. Many Turkish respondents had a low educational level. The respondents’ educational level corresponded reasonably to that of the general Dutch and Turkish population in the Netherlands [37].

**Table 2:** Characteristics of respondents

	**Total**		**Dutch**		**Turkish**	
	**n**	**%**	**n**	**%**	**n**	**%**
Sex of the child						
female	432	49.2	306	49.0	126	49.8
male	446	50.8	319	51.0	127	50.2
Child’s age						
6-8 years	466	54.3	340	54.8	126	52.9
≥ 8 years	392	45.7	280	45.2	112	47.1
Ethnicity						
Dutch	626	71.0	-	-	-	-
Turkish	256	29.0	-	-	-	-
Overweight child						
no overweight	643	84.3	506	89.9**	137	68.5
overweight	90	11.8	45	8.0**	45	22.5
obese	30	3.9	12	2.1**	18	9.0
Sex of the parent						
female	773	88.2	574	91.8**	199	79.3
male	103	11.8	51	8.2**	52	20.7
Parental education						
low	322	37.9	168	27.2**	154	66.4
middle	346	40.8	284	46.0**	62	26.7
high	181	21.3	165	26.7**	16	6.9

* p < 0.05, ** p < 0.01.

### Beliefs about overweight prevention and management

At univariate level, Dutch parents had stronger intentions to monitor outdoor play and snack intake, and they observed more outdoor play and lower snack intake in their child, compared to Turkish parents (Table 3). The belief that overweight is caused by genetic factors was held significantly more often by Turkish parents than by Dutch parents. Parental beliefs about genetic factors correlated negatively but weakly at a univariate level with intention to monitor outdoor play (r = -0.086, p < 0.05), meaning that parents who thought that genetic factors play a role were less inclined to be alert to outdoor play frequency. No significant association was found between beliefs about genetic causes of overweight and the intention to monitor snack intake.

**Table 3:** Parental beliefs about overweight prevention or management in their children and reported children’s behaviour per ethnic group (means and standard deviations (sd))

	**Total**	**Dutch**	**Turkish**
	**Mean (sd)**	**Mean (sd)**	**Mean (sd)**
Parental intention outdoor play (-2 = low; 2 = high)	.62 (1.24)	.76 (1.15)**	.27 (1.36)
Parental intention snack intake (-2 = low; 2 = high)	.98 (1.62)	1.12 (1.10)**	.62 (1.24)
Child behaviour playing outdoors (0 = never; 35 = all days more than 3 hours)	16.05 (9.16)	16.14 (8.44)**	15.81 (10.85)
Child behaviour snack intake (0 = never; 21 = all days more than 4 portions)	7.54 (5.95)	7.16 (5.99)**	8.50 (5.77)
Belief that genetic factors cause overweight (-2 = not important; 2 = important)	-.15 (.52)	-.18 (.47)**	-.07 (.63)

* p < 0.05, ** p < 0.01.

The intention to monitor snack intake was moderately correlated to the scale monitoring unhealthy eating behaviour from the Child Feeding Questionnaire (r = 0.39, p < 0.01), indicating an association between the parents’ monitoring intention and behaviour [33], In contrast to that, the association between the child’s outdoor play and snacking behaviour and the parent’s intention to monitor the behaviours was weak (for playing outdoors r = 0.086, p < 0.05 and for snacking r = -0.068, n.s.). The low correlations indicate that the intention of the parent and the actual behaviour of the child are two distinct features in the process of overweight prevention and management in children.

### Factors associated with parental intentions to monitor physical activity and diet

In the multivariate analyses, ethnicity was still significantly related to the parental intention to monitor the outdoor play and snack intake of the child after controlling for other distal factors and also after controlling for TPB-factors (β = -0.399, p < 0.01 and β = -0.373, p < 0.01 respectively in step 2, Table 4). In both models, the inclusion of the TPB determinants attitude and perceived social norms contributed substantially to parental intentions (change of R^2^ of 0.242 and 0.252 in step 2). Perceived behavioural control contributed significantly to the model with parental intention to monitor outdoor play as outcome, and not with intention to monitor snack intake.

**Table 4:** Multivariate models for parental intentions to monitor outdoor play and snack intake (standardised betas)

	**Outdoor play**			**Snack intake**		
	**intention**			**intention**		
	**Step 1**	**Step 2**	**Step 3**	**Step 1**	**Step 2**	**Step 3**
***Step 1: Distal factors***						
Ethnicity (Turkish vs. Dutch (reference)	-.466**	-.399**	-.399**	-.410**	-.373**	-.334*
Educational level (middle vs low (reference))	.213*	.128	.117	.336**	.171*	.175*
Educational level (high vs low (reference))	.137	.016	.006	.340**	.107	.122
Age of child	-.066**	-.047**	-.043**	-.066**	-.033*	-.032*
Perceived child weight (-2 = far too heavy, 2 = far too light)	-.315**	-.315**	-.436**	-.291**	-.236**	-.500**
Belief that genetic factors cause overweight (-2 = not important; 2 = important)	-.149	-.052	-.050	-.055	.015	.011
***Step 2: TPB factors***						
Attitude		.522**	.393**		.524**	.506**
Subjective norm		.366**	.432**		.386**	.407**
Perceived behavioural control		.194**	.242**		.079	.083
***Step 3: Interaction terms***						
Perceived child weight x attitude			.149			.248*
Perceived child weight x subjective norm			-.100			.071
Perceived child weight x perceived behavioural control			.050			.080
Ethnicity x attitude			.333			-.034
Ethnicity x subjective norm			-.167*			-.105
Ethnicity x perceived behavioural control			-.166			.022
ΔR^2^	.081	.242	.014	.096	.252	.012
Full model	R^2^ = .337			R^2^ = .360		

* p < 0.05.

** p < 0.01.

An interaction effect was found between subjective norm and ethnicity for intention to monitor outdoor play, and between attitude and perceived child weight for intention to monitor snack intake (step 3). The relationship between social norm and intention to monitor outdoor play was stronger in Dutch parents than in Turkish parents. The association between attitude and intention to monitor snack intake was stronger in parents who perceived their child’s weight as high, than in parents who believed that their child was of average weight. This association was absent in those who perceived their child’s weight to be low. The BMI SDS of the child was not significantly correlated with the outcome measures at univariate level and was therefore not included in the multiple regression analyses.

### Factors associated with reported child behaviour regarding physical activity and diet

Ethnicity was significantly associated with the outdoor play of children when controlled for other distal factors and TPB factors, whereas the relationship with snack intake was not significant (β = -0.057, p < 0.05 and β = 0.049, n.s. in step 2, Table 5).

**Table 5:** Multivariate models for the children’s outdoor play and snack intake behaviours (standardised betas)

	**Outdoor play**			**Snack intake**		
	**behaviour**			**behaviour**		
	**Step 1**	**Step 2**	**Step 3**	**Step 1**	**Step 2**	**Step 3**
***Step 1: Distal factors***						
Ethnicity (Turkish vs. Dutch (reference))	-.081**	-.057*	-.068	.092**	.049	.032
Educational level (middle vs low (reference))	-.031	-.014	-.015	-.017	-.031	-.026
Educational level (high vs low (reference))	-.042	-.014	-.014	-.026	-.023	-.018
Age of child	.005	.010**	.011**	.014**	.009	.009
Perceived child weight (-2 = far too heavy, 2 = far too light)	.008	-.007	-.014	.007	.025	-.015
Belief that genetic factors cause overweight	.001	.018	.016	.015	.010	.008
***Step 2: TPB factors***						
Attitude		.014	.002		-.082**	-.078**
Subjective norm		.001	-.003		.033 **	.033*
Perceived behavioural control		.144**	.147**		-.054**	-.058**
***Step 3: Interaction terms***						
Perceived child weight x attitude			.024			.005
Perceived child weight x subjective norm			-.017			-.015
Perceived child weight x perceived behavioural control			-.008			.050*
Ethnicity x attitude			.037			-.021
Ethnicity x subjective norm			.022			.000
Ethnicity x perceived behavioural control			-.007			.034
ΔR^2^	.018	.200	.006	.036	.049	.011
Full model	R^2^ = .224			R^2^ = .096		

* p < 0.05.

** p < 0.01.

In the model with the outcome outdoor play of the child, the contribution of the TPB factors was relatively high (change of R^2^ of 0.20), which was mainly explained by the parents’ perceived behavioural control of the outdoor play behaviour. The explained variance of the model with the outcome snack intake was small (R^2^ = 0.049), although all TPB factors turned out to be significantly related to the child’s snack intake.

An interaction effect was found between perceived behavioural control and perceived child weight (step 3). The association between the parent’s feeling to have control over the snack intake of the child and the reported snack intake was stronger in parents who perceived their child’s weight as high, than in parents who believed that their child was of average or low weight.

## Discussion

Ethnicity contributes to overweight related preventive behaviour for almost all outcome measures of this study. The outdoor play of Turkish children is less and snack intake is higher compared to native Dutch children. This is as expected, considering the large difference in prevalences of overweight and obesity in these ethnic groups. Dutch parents have stronger intentions to monitor outdoor play and snack intake than Turkish parents. Remarkably, for the child’s snacking behaviour an association with ethnicity was absent when controlled for the parental cognitive TPB factors. The TPB determinants are relevant for all the physical activity and diet outcomes of this study. With regard to the parental intention to monitor the child’s outdoor play and snack intake, attitude and social norm are the main contributing factors. With regard to the child’s outdoor play behaviour, the parent’s perceived behavioural control is an important contributor. For the child’s snacking behaviour, perceived behavioural control is a significant factor, especially in the group of parents who consider their child as overweight.

As most associations between the TPB-factors and overweight related preventive behaviour are not modified by ethnicity, it may be assumed that a deep structure in the cultural and psychological processes influencing overweight related behaviour specific for the ethnic groups is absent [28]. Only in one instance, effect modification by ethnicity was found. The intention of Turkish parents to monitor outdoor play is less dependent on the perceived social norm than in Dutch parents. In the qualitative interviews prior to this study mothers of Turkish children indicated that they received little support from their family in promoting overweight preventive behaviour, which may explain their independent position regarding their child’s outdoor play (unpublished data). An earlier study of dietary behaviour in ethnic groups also found an absence of cultural differences in parental perceived barriers or self-efficacy [25]. Furthermore, the parents’ beliefs about the role of genetic factors in overweight do not contribute significantly to the parental intentions and child’s behaviours with regard to physical activity and diet.

The inclusion of TPB determinants explained the largest proportion of variance in the regression models, however the sizes of theses proportions are still limited. Especially more insight is needed into factors determining the child’s snacking behaviour. Literature shows that parenting styles and environmental factors, that were not included in this study, are also relevant for obesity prevention [19-21,38]. Moreover the child’s individual attitudes with regard to playing outdoors and snacking will play an increasing role when they grow older. These factors may add to the understanding of the child’s snacking behaviour.

Although overweight and obesity were three times as high in the children of the Turkish response group, parental beliefs in this group about overweight prevention and management did not reflect a sense of urgency about changes in their child’s behaviour. A number of other studies in native and ethnic minority children of normal weight and overweight found that parents underestimate their children’s weight and are relatively unconcerned about childhood obesity [16,17]. In this study, child overweight status, as established objectively with weight and height scores (BMI) was also not important in terms of parents’ intentions to prevent or manage overweight, but parents’ subjective perceptions relating to a child’s weight were.

A limitation of this study is the cross-sectional study design. A longitudinal design could shed more light on the causal determinants of child behaviour and parental intention. The difference between the Dutch native and one large ethnic minority group is examined, which limits the generalizability of this study. However we were interested in relevance of cultural aspects for dietary and physical activity behaviour in general. Further testing of the relationship with behavioural cognitions in other minority groups is advised. Another limitation of this study is that questions were asked about parental intentions to encourage their child to play outdoors or monitor snack intake, while we asked for their child’s actual behaviour. The moderate correlation in our study between the intention to pay attention to the snack intake and the scale monitoring unhealthy eating behaviour from the Child Feeding Questionnaire is an indication of an association between parental intention and their monitoring behaviour. We were not familiar with a similar validated instrument on parental monitoring behaviour of the child’s physical activities, therefore actual parental monitoring behaviours were not measured. A third limitation may be the influence of the order of items and scales of the questionnaire on the respondents’ answering on certain types of questions, such as on perceived child weight and the child’s behaviour. These questions were placed at the beginning of the questionnaire to prevent answers influenced by preceding questions as much as possible, however possible response effects as a result of question order were not systematically pre-tested in this study. A final limitation of this study is its restriction to outdoor play and snacking behaviour. Other overweight related preventive behaviours, such as daily breakfast or sports participation, were not examined.

A strength of this study is the inclusion of an ethnic minority population in which parents of overweight children are overrepresented. This enabled us to explore beliefs and intentions in a population faced with a high prevalence of overweight children. The characteristics of the Dutch and Turkish response groups did not differ from the general populations in terms of educational level of the parents, and overweight and obesity, indicating the representativeness of our study results for these populations. The BMIs of the children of non-respondents could not be compared with those of respondents since we obtained informed consent to use register data from respondents only, and not from non-respondents.

The instruments and scales used in this study to measure intentions and behaviours were tailored to the ethnic minority response group using results from qualitative interviews. This meant that the number of items had to be limited, but the words and expressions used in the questionnaire are familiar to the Turkish respondents. The further development of robust instruments and scales for ethnic minority groups is recommended.

Several implications of the study results for preventive practice can be mentioned. Special attention has to be paid to the ethnicity of the target population when addressing parental overweight related preventive behaviours, given the differences between ethnic groups with regard to the behavioural outcomes of this study. The finding in literature that it is important to use a cultural approach to prevention, appropriate for norms and customs of the ethnic group, also applies to overweight and obesity behaviour [15,28,29]. Adaption of interventions to places and locations where the ethnic groups can be found, language, values and norms with regard to food quality and intake, and other cultural aspects are important to improve their reach among the targeted audiences [28]. As for most outcomes no interaction could be found between TPB-factors and ethnicity, it is hypothesized that preventive interventions targeting parents who contemplate or intend to change their behaviour, may follow a general theory-based approach to the social norms and attitudes with regard to overweight prevention or management in children, regardless of the ethnicity of the target population. However, for the intention to monitor outdoor play, the perceived social norm in Turkish parents could be strengthened, which implies that the perceived lack of support from the family with regard to overweight prevention has to be targeted also. When change of the actual behaviour of the child is aimed, the parent’s perceived behavioural control with respect to monitoring the physical activity and diet of the child appears an option for intervention. Furthermore, educational programmes and screening programmes should take into account the parent’s perception of a child’s weight status. Further research is advised into behavioural determinants regarding children’s dietary behaviour. Moreover, more insight has to be gained into behavioural determinants of dietary and physical behaviours in other ethnic groups. It is generally recommended that interventions to prevent overweight and obesity should start at an early child age. Our finding that parents intend to monitor physical activity and eating behaviour when the child is still young supports this recommendation.

## Conclusions

Native Dutch children and their parents show more favourable overweight related preventive behaviours than Turkish parents and children. As overweight related preventive behaviours and beliefs of both children and parents differ between the native and ethnic minority populations, it is advised that interventions pay attention to cultural aspects of the targeted population. Moreover, it is advised that interventions aimed at changing parental behaviours follow a uniform, theory-based approach, irrespective of the origin of the target population. Further research is recommended into parental behavioural cognitions regarding overweight prevention and management for different ethnicities.

## Keypoints

A comprehensive study of the relationship between behavioural cognitions of parents of pre-adolescent children and overweight preventive behaviour is lacking.

Differences have been found between ethnic populations regarding the children’s physical activity and diet behaviours and parental intentions to monitor these behaviours.

Parental attitudes and social norms contribute to the intention to monitor the child’s outdoor play and snack intake.

Parental perceived behavioural control is associated with the child’s outdoor play and, in parents who perceive their child to be overweight, with the child’s snacking behaviour.

## Competing interests

The authors declare that they have no competing interests.

## Authors’ contributions

PK was responsible for protocol development, data collection, data analysis, the drafting of the manuscript and supervision of the execution of the study. YS collected and analysed the data and contributed to the writing of the manuscript. LH and CJ participated in the development of the study protocol and contributed to the drafting of the manuscript. SD had the primary responsibility for the development of the study protocol, supervised the execution of the study with PK, and contributed to the drafting of the manuscript. All authors read and approved the final manuscript.

## Pre-publication history

The pre-publication history for this paper can be accessed here:

http://www.biomedcentral.com/1471-2458/12/867/prepub
